# Enhanced Hydrophobicity of Polymers for Protective Gloves Achieved by Geometric, Chemical and Plasma—Surface Modification

**DOI:** 10.3390/ijerph19095239

**Published:** 2022-04-26

**Authors:** Emilia Irzmańska, Mariusz Siciński, Aleksandra Smejda-Krzewicka, Agnieszka Adamus-Włodarczyk, Magdalena Makowicz, Tomasz Gozdek

**Affiliations:** 1 Department of Personal Protective Equipment, Central Institute for Labour Protection—National Research Institute, 16 Czerniakowska, 00-701 Warsaw, Poland; agada@ciop.lodz.pl (A.A.-W.); makowicz.m.95@gmail.com (M.M.); 2 Institute of Polymer and Dye Technology, Lodz University of Technology, 16 Stefanowskiego, 90-537 Lodz, Poland; mariusz.sicinski@p.lodz.pl (M.S.); aleksandra.smejda-krzewicka@p.lodz.pl (A.S.-K.); tomasz.gozdek@p.lodz.pl (T.G.)

**Keywords:** PPE, gloves, surfaces modification, hydrophobicity, polymers

## Abstract

Gloves are one of the most important elements of personal protective equipment (PPE). To improve gloves properties, a lot of different methods of surface modifications are used. In this work, the application of geometric, chemical, and plasma surface modifications to improve the hydrophobicity of butyl (IIR) and silicone (MVQ) rubber are described. To characterise surface properties contact angle measurements, FT-IR spectroscopy and scanning electron microscopy were used. This study showed that when the chemical modification applied, the contact angle value increases compared to non-modified samples. In addition, plasma modification raised the contact angle value and smoothed the surface morphology. An increase in the polymer surfaces hydrophobicity was the observed effect of the three modifications of rubber.

## 1. Introduction

Polymer protective gloves represent a significant material group of personal protective equipment (PPE). Nowadays, protective gloves are made of natural rubber, acrylonitrile-butadiene rubber, butyl rubber, polyvinyl chloride, and chloroprene rubber latex [1,2,3]. The gloves are meant to protect their users’ hands from harmful factors, but their comfort—which also improves safety—is an equally vital aspect. Considering the safety of using polymer gloves, particularly in a contaminated and humid environment, good wettability and hydrophobicity are essential because they enable effective draining of hazardous and harmful chemicals away from the glove [4,5]. Hydrophobic materials are characterised by a contact angle greater than 90°, whereas the contact angle of superhydrophobic surfaces exceeds 150° [6,7,8]. Moreover, highly hydrophobic surfaces are characterised by low values of free surface energy [9].

In order to obtain a material with surface hydrophobicity higher than in standard materials used currently for protective gloves, surface geometrisation can be used. Inspiration with natural structures marks an interesting search direction for concepts of geometric patterns promoting material’s hydrophobicity [10,11]. The attempts to represent the hierarchic structure of a lotus leaf are the best-known examples of imitating nature; the structure’s discovery triggered the research stream related to superhydrophobic surfaces [12,13]. The latest literature findings indicate that obtaining superhydrophobic surfaces on smooth surfaces is extremely difficult and highly unlikely. In order to obtain materials characterised by a contact angle greater than 150°, the material’s surface needs to be modified to increase the surface roughness [14,15]. The materials whose structure contains micro- and nanoprotrusions are characterised by lower capillary forces on the liquid/air border, facilitating the liquid’s drop flowing down the material’s surface. Therefore, contaminants are easily removed.

The application of chemical modifications (e.g., wet chemical etching, sol-gel, and chemical vapour deposition) and physical modifications (laser ablation and plasma surface treatment) is another direction used in making superhydrophobic surfaces [13,14,15].

Polymer materials used for protective gloves reveal different surface wettability. The authors of this paper decided to use IIR butyl rubber to represent the group of materials currently used for making polymer protective gloves. The material was selected due to its hydrophobicity (contact angle of ca. 90°) in the primary condition and a broad range of possible modifications. Butyl rubber is a non-polar elastomer with a very low double bond content. Therefore, products made of it are characterised by excellent chemical resistance, as they are resistant to acids, alkalis, ozone, oxygen, and elevated temperature, which is of great importance in the production of protective gloves exposed to the above-mentioned reagents. In addition, IIR materials are characterised by a very low vapour and gas permeability, which is an additional advantage. Therefore, protective gloves manufactured from IIR could be used in many industrial applications. MVQ silicone rubber was selected as the other material; nowadays, it is not used for protective gloves. Still, its properties, such as good elasticity and resistance to oxidation, water vapour and some chemicals, make it a good candidate material for polymer protective gloves. Physiological harmlessness constitutes its great advantage, making the material suitable for direct contact with food and, hence, safe. MVQ is an elastomer resistant to mould and bacteria, and is characterised by high and selective vapour and gas permeability (in contrast to IIR); therefore, its cross-linked products can be successfully used in numerous medical applications. Silicone rubber can be well used for materials intended for Personal Protective Equipment [16].

The authors of the publication also described the effect of physical modification in the form of laser ablation. They presented promising results in the publication.

However, due to the costly use of laser ablation in the technological process, plasma modifications have been proposed. This modification allows the device to be used directly on the processing line. The process is also less time consuming than laser ablation.

Surface modification using plasma has become a popular method of making hydrophobic surfaces. The method’s main advantages include the possibility of controlling the process parameters, environmental friendliness, and a broad application range [17,18]. Gao et al. used CF4 RF capacitively coupled plasma to improve the silicone rubber’s hydrophobicity; the plasma power ranged from 60 to 200 W, at the 20 sccm flow for 20 min [19]. Vazirinasab et al. used 21 kHz atmospheric-pressure plasma, at the nozzle-to-sample distance of 8 mm, to produce micro- and nanoprotrusions on the silicone rubber surface to improve its hydrophobicity [20]. The above parameters helped obtain a surface with a contact angle >160°. Cho et al., in their paper, described the method based on plasma etching to improve the hydrophobicity of silicon surfaces [21]. The authors used inductively-coupled plasma (ICP) and SiO_2_ shields and performed alternating etching and depositing to obtain a hierarchic structure on the silicon surface, composed of superhydrophobic pins (contact angle > 160°). During etching, the source’s power and polarisation voltage amounted to 800 W and −50 V, while at the deposition stage, the source’s power remained unchanged, but the voltage was 0 V. Di Mundo et al., in their paper, used plasma etching combined with a silicone film deposition on polycarbonate substrates to produce superhydrophobic non-uniformly rough surfaces [22]. The authors used a plasma reactor with the input power of 50–200 W, with the O_2_ flow at 10 sccm and 13.3 Pa pressure to perform the process. Kontziampasis et al. used plasma nanotexturing/etching in an oxygen atmosphere to modify PMMA surface to provide anti-reflective material properties [23]. Texturing was carried out at the following parameters: polarisation generator power, 250 W; source power, 1800 W; temperature, 15 °C; etching time range, 30–180 s; and etching rate, 1400 nm/min. The conditions helped obtain the surface’s wettability angles of about 150°.

The combination of three modifications—geometrical, chemical, and physical modification based on plasma—can improve the wettability of polymer surfaces. Chemical methods of improving the material surface polarity are typically used for making hydrophobic protective layers. There are also proven methods of making polymer gloves with a knurled (geometrised) layer with a convex pattern in the macro scale. Their contact angle amounts to ca. 110°.

This paper describes the application of geometric, chemical, and plasma surface modifications to improve the hydrophobicity of cured rubber. The tests were carried out on MVQ silicone rubber and IIR butyl rubber.

## 2. Materials and Methods

### 2.1. Preparation of Samples

The samples described in the paper were made of butyl rubber (IIR) from ExxonTM Chemical (Houston, TX, USA) and silicone rubber (MVQ) provided by Zakład Chemiczny “Silikony Polskie” Sp. z o.o. (Nowa Sarzyna, Poland).

Table 1 and Table 2 summarise the compositions of the produced rubber mixes.

The rubber composites were prepared using a Krupp–Grusan laboratory two-roll mill with a roll diameter of 200 mm and 450 mm long. The temperature of the roll was 25–40 °C, while the speed of the front roll was 20 rpm, with the roll friction of 1:1.25. The first step of making the mixes involved rubber plasticising for 30 s. Then, other ingredients were introduced in the following sequence:

(a) For the IIR mix: stearic acid, zinc oxide, Arsil and paraffin oil, MBT, and TMTD. Then, the cross-linking substance (sulphur) was introduced and the whole system was homogenised. The mix preparation time was 12 min;
(b) For the MVQ mix: fine dicumyl peroxide and Aerosil. Then, the whole system was homogenised. The mix preparation time required for thorough mixing of all its ingredients amounted to 14 min.

The material was cured and subjected to geometric, chemical, and physical modifications at the next step.

### 2.2. Geometric Modification

**Figure 1:**
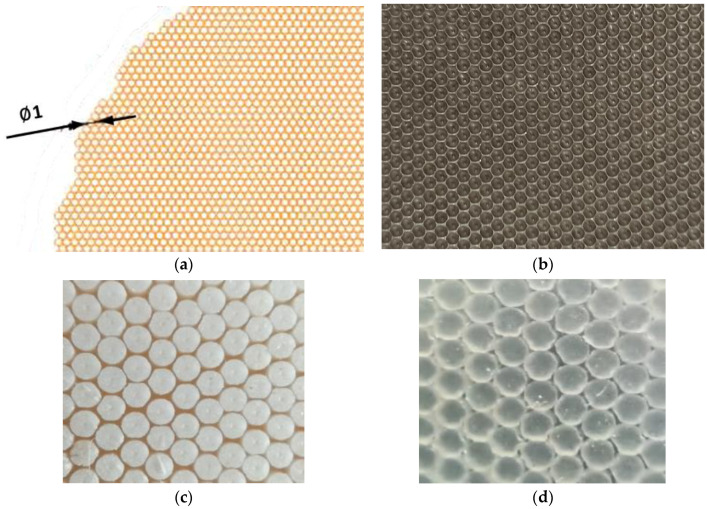
(**a**) Design of a geometrised aluminium mould; (**b**) actual geometrised aluminium mould used in curing; (**c**) actual sample made of butyl rubber; and (**d**) actual silicone rubber sample.

During curing, geometric modification of the produced rubber mixes was carried out using an auxiliary geometrised matrix placed in moulds as a spacer. The template used in geometrisation consisted of flat-bottom holes, Ø 1 mm diameter and 0.8 mm deep. The matrix was made with a CNC machine. The design and the actual matrix are shown in Figure 1.

The setup scheme for the measurements is provided in the Figure 1.

The produced rubber mixes were cured in metal moulds placed between the heated shelves of the hydraulic press. The mixes were protected with Teflon foil. In order to produce polymer materials with geometrised surfaces, a spacer shown in Figure 1b was placed inside the metal moulds. The pre-heated spacer was sprayed with an anti-adhesive agent (McLube 1725E, McGEE Industries Inc., Aston, PA, USA—for butyl rubber mix, McLube MAC 968, McGEE Industries Inc.—for silicone rubber mix) to facilitate separating the geometrised material from the mould. The rubber mixes were cured at 160 °C, 150 bar pressure for a period determined in vulcametric curves. The curing time of the mix amounted to 10 min. The samples prepared with this method are marked as G further in the paper.

### 2.3. Chemical Modification

The next step involved chemical modification of the obtained smooth and geometrised samples. The surface modification involved immersing polymer samples in a 10% bis[(3-triethoxysylil)propyl]tetrasulfide solution in toluene for 10 min. Finally, the samples were placed in an incubator at 45–50 °C to dry. The chemically modified samples are marked as C further in the paper.

### 2.4. Plasma Modification

**Figure 2:**
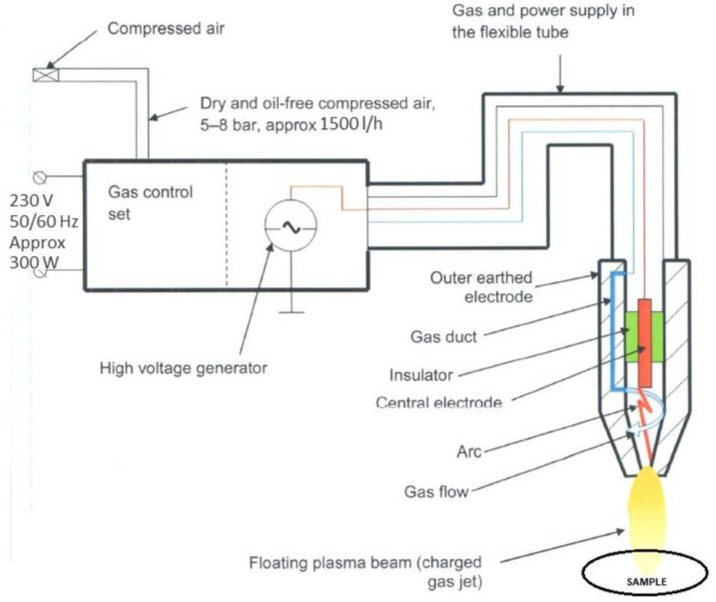
Apparatus used for plasma modification processes—illustrative diagram.

**Figure 3:**
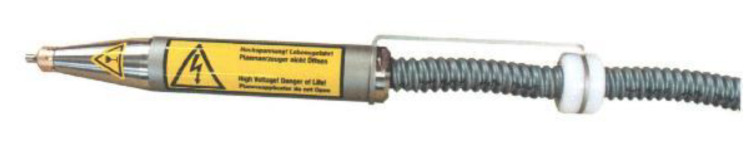
Nozzle used for plasma functionalisation.

The polymer samples were plasma-modified with Diener apparatus, Plasma Beam Standard model (Germany), with 300 W power and 10 kV generator voltage. Two gases were used for the modifications: air and argon. Pressurised (5–8 bar) compressed air (cooling gas) was used. The processes were carried out at the feed rate of 5 cm/s and the beam travel stroke of 1.5 cm. The modified sample’s distance from the nozzle was 2 cm. Figure 2 shows a diagram of the equipment used. Figure 3 shows the nozzle used for the modification.

### 2.5. Contact Angle Measurements

The developed polymer surfaces were evaluated with a drop deposition method for wettability. Phoenix Alpha from SEO (Suwon, Korea) was used for the measurements. The measurement involved placing a deionised water droplet (10^−3^ cm^3^ volume) on the sample’s surface and taking its photo. The photos were analysed with Phoenix Alpha Contact Angle Analyzer software.

The measurements were performed for samples before and after plasma modification. Ten contact angle measurements were performed for each sample, and then the contact angle’s mean value was calculated.

### 2.6. Spectra Development with FT-IR Spectroscopy

Fourier transform infrared spectroscopy (FTIR) absorbance spectra of the GTR surfaces were collected in the 4000–400 cm^−1^ range (64 scans, resolution of 4 cm^−1^). The experiments were performed with a Nicolet 6700 FTIR spectrometer equipped with a Smart Orbit ATR sampling accessory (Thermo Scientific, Waltham, MA, USA) operating with a diamond crystal.

### 2.7. Surface Morphology Observations with Scanning Electron Microscopy (SEM)

The cross-section and morphology of the polymer samples were examined with the Tabletop Microscope TM-1000 scanning electron microscope from Hitachi (Tokyo, Japan). A gold film was deposited on the samples before the test using the Sputter coater 109 auto vacuum spraying system from Cressington (Redding, CA, USA) under 40 mBa pressure for 60 s. The SEM images of the samples were analysed with TM-1000 software (Ramsay, NJ, USA) at 100× magnification.

## 3. Results

### 3.1. Contact Angle Measurement Results

Table 3 summarises the mean values of the contact angle measurements for the produced samples: smooth and chemically and physically modified with two gases; the samples were made of IIR butyl rubber and MVQ silicone rubber.

**Figure 4:**
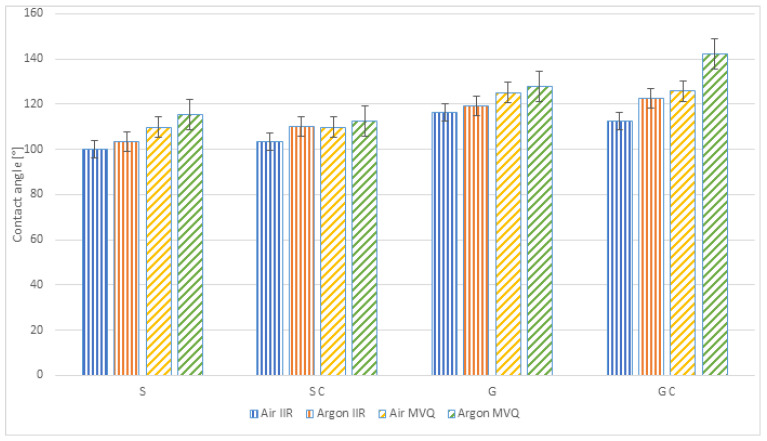
Contact angles of the analysed polymer materials (S—smooth samples, SC—chemically modified smooth samples, G—geometrised samples, GC—chemically modified geometrised samples; Air IIR—samples of IIR butyl rubber polymer materials subjected to plasma modification in the air; Argon IIR—samples of IIR butyl rubber polymer materials subjected to plasma modification in argon; Air MVQ—samples of MVQ silicone rubber polymer materials subjected to plasma modification in the air; Argon MVQ—samples of MVQ silicone rubber polymer materials subjected to plasma modification in argon).

The results are presented in a graphic form in Figure 4 including standard deviations (marked).

### 3.2. Results of Structure Analysis with IR Spectroscopy

**Figure 5:**
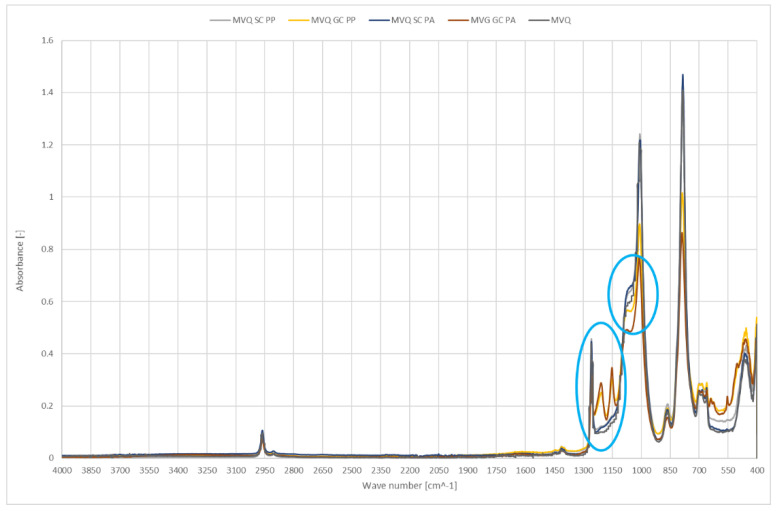
IR spectra of polymer materials made of silicone rubber (SC—chemically modified smooth samples, GC—chemically modified geometrised samples subjected to plasma modification in the air (PP) or argon (PA), and for non-modified polymer material made of silicone rubber).

Figure 5 shows IR spectra of smooth and geometrised samples made of silicone rubber; the samples were chemically- and plasma-modified. Polymer material made of chemically modified silicone rubber was the reference sample.

**Figure 6:**
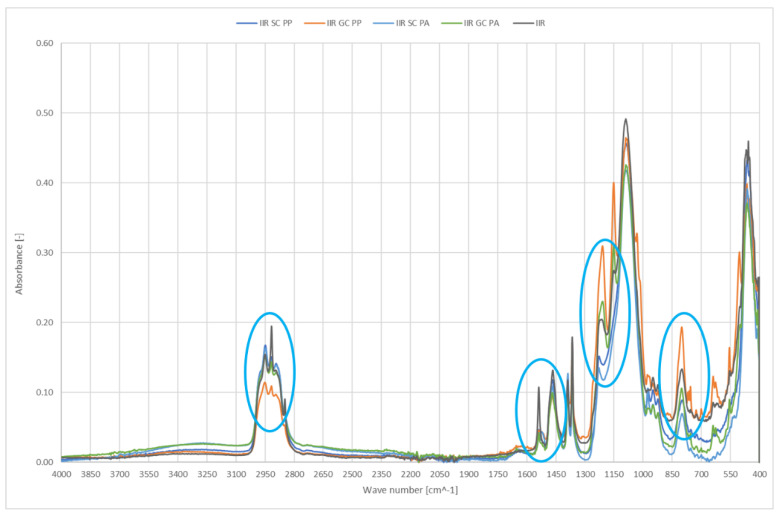
IR spectra of polymer materials made of butyl rubber (SC—chemically modified smooth samples, GC—chemically modified geometrised samples subjected to plasma modification in the air (PP) or argon (PA), and for non-modified polymer material made of butyl rubber).

Figure 6 shows IR spectra of smooth and geometrised polymer samples made of chemically modified butyl rubber subjected to plasma modification. Chemically modified butyl rubber was the reference sample.

### 3.3. Results of SEM Observations of the Surface Morphology

**Figure 7:**
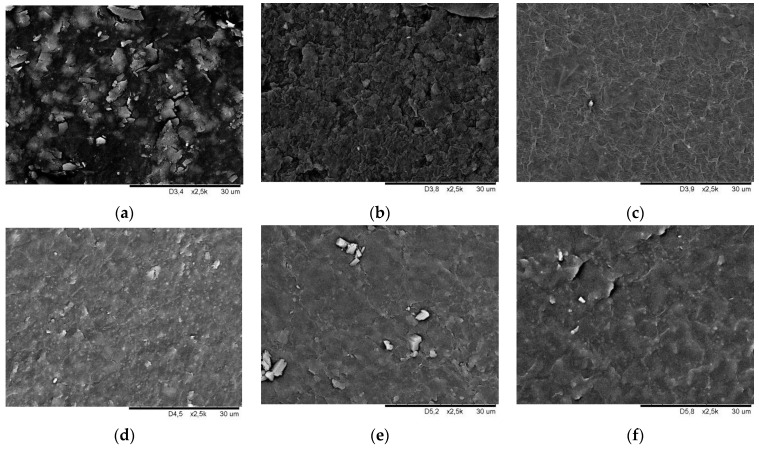
Surface morphology SEM image of butyl rubber smooth samples (**a**) chemically non-modified, (**b**) plasma-modified in the air, (**c**) plasma-modified in argon, (**d**) chemically modified, (**e**) chemically and plasma-modified in the air, and (**f**) chemically and plasma-modified in argon.

**Figure 8:**
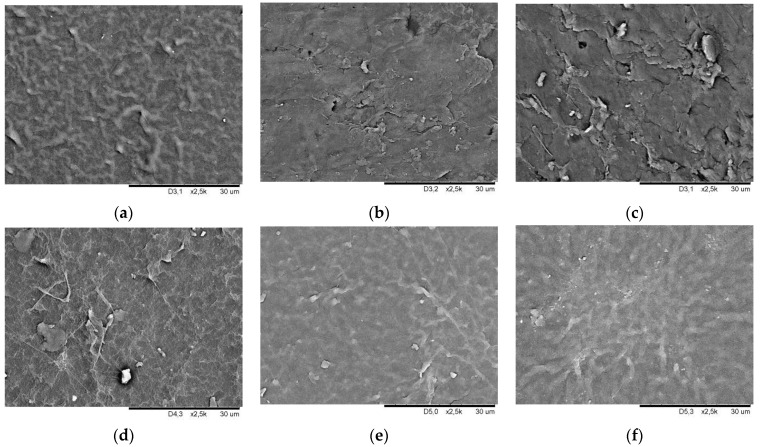
Surface morphology SEM image of silicone rubber smooth samples (**a**) chemically non-modified, (**b**) plasma-modified in the air, (**c**) plasma-modified in argon, (**d**) chemically modified, (**e**) chemically and plasma-modified in the air, and (**f**) chemically and plasma-modified in argon.

Figure 7 and Figure 8 show SEM images of smooth and geometrised samples’ surfaces for non-modified and plasma-modified samples made of silicone and butyl rubber.

## 4. Discussion

Protective materials for polymer gloves, with a set hydrophobicity, enabling effective draining of hazardous and harmful chemicals away from the glove in the work environment, were the subject of the study [24]. The study used a combination of geometric, chemical, and physical modification for MVQ silicone rubber and IIR butyl rubber samples. Combining three different modification methods of the rubber surfaces enabled improving the tested materials’ hydrophobicity.

Higher contact angle values before plasma modifications were obtained for silicone rubber samples, as shown in Figure 4. For the MVQ, the contact angle values amounted to 98°, while their value for the IIR was 90°. Plasma modification, using air and argon, increased the contact angle value for both types of rubber. For the IIR, the increase from 90° was observed by 10% for plasma using air and by 15% when argon was used. For the MVQ, a higher increase was observed—by 18% and 22%, respectively.

The performed modifications revealed that the highest contact angle value was obtained for the polymer material made of MVQ rubber geometrically, chemically and physically modified in argon (142.12°). For the non-modified, smooth samples, a tendency can be observed of the contact angle increase after each modification, but the highest contact angle values, both for the MVQ and IIR polymer materials, were obtained after plasma modification in argon. Comparing the analysed materials, one can observe that better hydrophobic properties characterised samples made of MVQ silicone rubber.

The contact angle (water) and wettability of the plasma-treated surface depend on the surface roughness combined with the substrate’s chemical composition. The following two changes occur in polymer substrate during plasma treatment: (1) chemical modification through the surface reaction with plasma forms to generate new functional groups, and (2) physical etching through chemical reaction and surface etching leading to surface roughness [25]. Since silicone rubber chains contain organic groups (such as methyl) linked to an inorganic siloxane matrix, organic groups are more susceptible to etching during air plasma treatment because of oxygen reaction with carbon and hydrogen.

E. Vazirinasab et al. used SEM analysis to observe roughness formation and surface micro- and nanomorphology of the plasma-treated silicone rubber surfaces, modified at different plasma treatment voltages. Before plasma treatment, the silicone rubber’s surface was relatively smooth, whereas after plasma treatment at 80% voltage, nanometric protrusions were observed. The protrusions became more evident at higher voltages, and coral-like structures dominated the morphology. The cluster sizes and coalescence increased at higher plasma voltages, which reduced the spaces between the clusters and developed microroughness on the substrate [20].

E. Vazirinasab et al., in the FTIR analysis, observed gradual elimination of hydroxy groups and a rising plasma voltage as a result of decomposition of alumina trihydrate fillers under high temperatures of plasma treatment.

In the tested materials, new peaks at the wavenumber of 1200 cm^−1^ were observed after geometrised surface modification; the peaks confirm the occurrence of new C-C bonds and a higher number of CH_3_ groups, which can be a testimony to shortening or rearrangement of polymer chains. An additional peak at 1150 cm^−1^ is the evidence of C-O groups occurrence in the structure. The reduced intensity in the 1100–900 range and 785 cm^−1^ results from a smaller number of Si-O-Si and Si-O in O-Si-CH_3_ groups, most likely related to regrouping in the polymer chain and disruption of the bonds in the rubber structure. Most importantly, the effect does not occur in plasma-treated smooth surfaces.

A geometrised sample’s modification in the air reduces the number of C-H groups, 3000–2800 cm^−1^. Plasma application cleans the surface of NH and N=O groups which can originate from additives; it can be observed by reduced peak intensity at 1540 cm^−1^. Increasing the band intensity for the wavenumber of 1200 cm^−1^ responsible for deforming vibrations of the OH group is proof of surface oxidation; the effect is observed only for geometrised samples, while air plasma causes many significant changes. A higher number of C-O-C groups at 800 cm^−1^ can also confirm oxidation of the sample’s surface exposed to oxygen plasma. Moreover, an increase in the number of C-C groups occurs in the samples with a larger specific surface, observed as stretching vibrations for 1150 cm^−1^; in this case, a more significant effect occurs for air plasma.

The photos show the morphology of non-modified and chemically modified butyl and silicone rubber smooth samples. A fuzzy surface was observed for non-modified butyl rubber, which became smooth, after plasma modification, with visible particles on the surface. Plasma modification using argon (Figure 7c) leaves surface irregularity and roughness, which is confirmed by the increase in the contact angle value compared to the results for plasma-treated samples using air (Figure 7b). Surface smoothing caused by 10% bis[(3-trietoxsylil)propyl]tetrasulfide solution in toluene was observed for the chemically-modified butyl rubber samples (Figure 7d). Plasma emphasises the surface irregularities, which become larger but are less densely distributed.

Surface cracking is observed for non-modified silicone rubber due to material degradation under plasma (Figure 8b,c). Similar to butyl rubber, the surface of chemically modified silicone samples becomes smoother. Plasma in the case of silicone rubber makes irregularities less evident (Figure 8e,f).

## 5. Conclusions

Silicone and butyl rubber materials used in the study were subjected to geometric, chemical, and atmospheric-pressure plasma modification. Selecting one modification method as the best is impossible because it is a process of chemical and physical modification that have been evaluated simultaneously. The contact angle of 142° was obtained for measurements using water, which is a very high value for protective polymer materials. The chemical modification increases the contact angle value compared to non-modified samples. In addition, plasma modification raised the contact angle value and smoothed the surface morphology. A growing contact angle trend was observed for the materials subjected to the three modifications for both types of rubber.

Our objective was to achieve higher contact angle values. An increase in the polymer surfaces hydrophobicity was the observed effect of the three modifications of rubber. Still, the effect contradicted the literature findings that report roughness occurrence on the rubber surface. Therefore, the optimum modification conditions should be developed to produce the best structure that will significantly increase the contact angle value.

## Figures and Tables

**Table 1:** Composition of rubber mixes containing butyl rubber (IIR).

Ingredient	Parts by Weight
Butyl rubber	100
Stearic acid, CHEMPUR, Piekary Śląskie (Poland)	1
Zinc oxide, CHEMPUR, Piekary Śląskie (Poland)	5
Sulphur, CHEMPUR, Piekary Śląskie (Poland)	2
2-mercaptobenzothiazole (MBT), ACROS ORGANICS, Geel (Belgium)	1
Tetramethylthiuram disulphide (TMTD), ACROS ORGANICS, Geel (Belgium)	1
Arsil silica 120 m^2^/g, Zakłady Chemiczne Rudniki S.A., Rudniki (Poland)	30
Paraffin oil	3

**Table 2:** Composition of rubber mixes containing silicone rubber (MVQ).

Ingredient	Parts by Weight
Silicone rubber	100
Dicumyl peroxide, Sigma Aldrich Co., Saint Louis, MO, USA	0.95
Aerosil silica 380, 380 m^2^/g, Evonik Industries AG Germany, Essen (Germany)	30

**Table 3:** Summary of the analysed materials’ contact angle values.

	Air	Argon
**Butyl rubber IIR**		
**IIR** non-modified	92.00	
**IIR S**	99.85	103.43
**IIR SC**	103.2	109.94
**IIR G**	116.33	119.33
**IIR GC**	112.57	122.68
**Silicone rubber MVQ**		
**MVQ** non-modified	99.811	
**MVQ S**	109.83	115.18
**MVQ SC**	109.78	112.6
**MVQ G**	125.15	127.72
**MVQ GC**	125.88	142.12

IIR—butyl rubber, MVQ—silicone rubber, S—smooth samples, SC—chemically modified smooth samples, G—geometrised samples, GC—chemically modified geometrised samples.

## Data Availability

Not applicable.
